# Leading through the COVID‐19 pandemic: Experiences of UK Executive Nurse Directors

**DOI:** 10.1111/jan.16329

**Published:** 2024-07-14

**Authors:** Alison H. James, Rebecca Dimond, Aled Jones, Dianne Watkins, Daniel Kelly

**Affiliations:** ^1^ School of Healthcare Sciences Cardiff University Wales UK; ^2^ School of Nursing and Midwifery Plymouth University Plymouth UK

**Keywords:** COVID19, director of nursing, executive nurse director, leadership, pandemic, qualitative research

## Abstract

**Aim:**

To explore the challenges experienced by Executive Nurse Directors during the COVID‐19 pandemic, and to inform future nursing leadership strategies.

**Design:**

A qualitative research project involving interviews with 21 Executive Nurse Directors from England and Wales.

**Methods:**

Participants were purposively sampled and recruited through Chief Nursing Officers and nursing leadership networks. Semi‐structured interviews were conducted and recorded online via Teams. Braun and Clarke's approach to thematic reflexive analysis was applied to data analysis.

**Results:**

Executive Nurse Directors played a critical role during the COVID‐19 pandemic. Six themes are explored: tensions, and adaptive response to personal leadership styles; uncertainty and support at the board level; responding to national political decision‐making; the personal and emotional impact of the role and the sources of effective support; the voice and public profile of nursing; lessons learnt and strategies for future leadership development. Enablers of decision‐making included effective multidisciplinary working, freedom from normal organizational constraints, support for innovation, and the development of stronger bonds with colleagues. Barriers to decision‐making included limited knowledge of the virus and its impact and lack of guidance, particularly at a national level. Priorities, strategies and actions for recovery include recognizing the emotional impact of being in a high‐level decision‐making role, protecting staff from burnout and understanding the long‐term implications of pandemic work for nurse leaders.

**Conclusion:**

Future strategies for nursing leadership during public health, national and global emergencies are recommended.

**Impact:**

This study contributes to the literature exploring the Executive Nurse Director role and their experiences of leading through the COVID‐19 pandemic, and identifies priorities, strategies and actions for recovery and learning for the future of senior leadership.

**Reporting Method:**

The study adhered to the Consolidated Criteria for Reporting Qualitative Research.

**Patient or Public Contribution:**

No patient or public contribution.

**Impact statement**

This study contributes to the literature exploring the Executive Nurse Director role and their experiences of leading through the COVID‐19 pandemic, and identifies priorities, strategies and actions for recovery and learning for the future of senior leadership. By understanding the experience of nurse leaders during the pandemic, this study identifies how nurse leadership can be better supported in the future and acknowledges the contribution and wealth of expertise that this level of nurse leader brings to the profession.

## 1 INTRODUCTION

Executive Nurse Directors (also known as Directors of Nursing) have lead responsibility and accountability at the executive board level including areas of patient safety, quality of care, staff well‐being, infection control and patient experience. These roles are identified globally as having unparalleled insight and responsibility for the quality of care provided within healthcare organisations (Jones et al., 2016; Kelly et al., 2023; World Health Organisation (WHO), 2020). Executive Nurse Directors have a unique perspective and overview of nurse leadership requirements and decision‐making, which can inform future strategies during times of health crises. This study aimed to explore the experiences of nurses working at the executive board level during COVID‐19, and to produce empirical evidence identifying effective nurse executive leadership strategies during this time, including how the role adapted in response. Recommendations for strengthening the nurse executive role in such situations in the future are provided. Learning from the experiences of Executive Nurse Directors during the COVID‐19 pandemic (referred to as ‘the pandemic’ hereinafter), and their strategies and priorities for recovery, is crucial to help inform and support effective global nursing leadership strategies at all levels in the profession (Maben & Bridges, 2020).

## 2 BACKGROUND

This study explores the experiences of the senior nurses working in the National Health Service (NHS) in England and Wales. We conducted a global literature search of databases including Pubmed, Scopus and CINAHL, using keywords, ‘director of nursing,’ ‘executive,’ ‘nurse leader’, ‘chief’ and ‘nursing leadership’. We then modified our search to include ‘pandemic,’ ‘covid’ and ‘coronavirus,’ SARS‐Cov‐2′. The search was initially conducted in March 2023, and updated in both July 2023 and September 2023 and included evidence from 2013 to 2023 to include pandemic evidence.

From the global literature, it is difficult to determine the overall contribution and effectiveness of the leadership of Executive Nurse Directors given the variation in roles and titles of executive‐level nurses, the healthcare contexts within which they work, as well as differing governmental responses to the pandemic within individual countries. No studies were found that directly explored the role of board‐level executive nurses with strategic responsibilities during the pandemic. Therefore, in conducting the literature review, a wider approach was taken to include international evidence of comparable, but not necessarily identical roles.

### 2.1 The complex role of executive nurse directors

The portfolio of Executive Nurse Directors is broad and varied depending on their organization and setting within the UK and internationally, including for example, ‘strategy, commissioning, organizational development, communications, patient and public involvement, marketing and facilities’ (NHS Improvement, 2019, p. 6). From the pre‐pandemic literature, Kelly et al. (2023) suggested that the Executive Nurse Director role is wide‐ranging and more multidimensional than any other member of the executive board. Jones et al. (2016) highlighted Executive Nurse Directors needed to be credible and competent, whilst forging effective working relationships with other board members. The role is complex and mirrors significant organizational and policy changes within health and social care. The brevity of tenure and high vacancy rate for the Executive Nurse Director roles pre‐pandemic indicates the challenging exigencies of the role and identified suggestions of lack of support and preparation (Kelly et al., 2016), raising the question of possible exacerbation of the issues caused by the pandemic. In their review of pre‐pandemic literature, Horseman et al. (2020) acknowledge the difficulties of reviewing the evidence for this group with no agreed definition of the role, and therefore its distinct remit is often not distinguishable from other nurse managerial roles. This, along with the unparalleled challenge of the pandemic, provided a clear focus for this study and the importance of exploring the Executive Nurse Directors experiences and perceptions of their leadership during the pandemic.

### 2.2 The role of Executive Nurse Directors during the pandemic

Executive Nurse Directors have prominent levels of responsibility and potential influence within health care organizations (Riddell et al., 2022). During the pandemic this involved overseeing extensive transformation and re‐organization of frontline services, which for some included rapidly establishing temporary hospitals, closure of existing departments and redeployment of staff, and organizing the vaccination delivery programme. Nurse leaders appear to have adapted to the difficulties of rapid decision‐making within ever‐changing and uncertain conditions, drawing on their leadership experience, whilst also taking risks in unchartered territories and enabling innovative and creative solutions (Riddell et al., 2022). Much of the broader evidence on the experiences of nursing during the pandemic focuses on frontline staff, reflecting greater numbers, yet with less strategic decision‐making and power. Less evidence exists on the higher level nurse leaders, driving the need to explore the distinct experiences of this elite group. The need to explore their perceptions also has prominence for future and aspiring nurse leaders and preparation for leading through future public health crises.

From the literature search, the mainly international evidence pointed to the importance of leadership skills during this crisis. Executive‐level nurse leaders' decision‐making strategies can be seen to have had both immediate and long‐term impacts on staff and patients (Lucas et al., 2022), and on public perceptions of the nursing profession (Wymer et al., 2021). Synthesizing the available evidence to understand and support nurse leaders comes at a critical moment for nursing. The effects of the pandemic on the global workforce have been linked with moral distress, depression and anxiety, staff absenteeism and a higher risk of nurse leaders leaving the profession, often without a clear succession plan in place (Couper et al., 2022; Foster, 2023; Miller et al., 2023).

Some studies have explored the support and preparation for the role of management, Holge‐Hazelton et al. (2021) compared the experiences of ward managers with heads of departments in Denmark and found those with more formal education in management, and those with greater personal experience, were more likely to consider themselves competent to manage during the pandemic. Also in Denmark, Morrison and Jensen (2022) interviewed 12 nurse managers and highlighted how accessing support and having a clear vision were significant factors in creating positive experiences for nurse leaders, enabling support of others, and offering protection from stress and conflict. Monroe et al. (2022) interviewed 9 Chief Nursing Officers in the United States of America (US) and identified significant emotional and administrative challenges, with nurse leaders feeling frustrated, exhausted, overwhelmed and lonely. Freysteinson et al. (2021) interviewed 28 US nurse leaders exploring how they had navigated the pandemic, their embodying of leadership and seeking ways to induce a sense of calm. Joslin and Joslin (2020) and the American Organization for Nursing Leadership surveyed and conducted additional conversations with US nurse leaders (1824 questionnaires). Respondents reported capability in meeting challenges, with emotional health and well‐being of staff being the most challenging, and the constant changes demanded by the pandemic posing tensions for the promotion of a trusting relationships between staff and patients. These findings focus on management roles and although characteristics of leadership are highlighted in some, the board‐level decision‐making and relationships are not the focus.

Further studies from the US include Losty and Bailey (2021) who interviewed 6 nurse executives exploring leadership and innovation during the pandemic. Communication, presence and mental toughness were considered the key components for quality care provision during challenging times. In a survey of various managerial roles Montgomery and Patrician (2022) found a supportive work environment for leadership was linked to lower levels of burnout resilience and higher levels of resilience and staff retention. Fowler and Robbins' (2022) survey of 70 nurses in differing roles found that senior nurses experienced a lack of trust and a sense of isolation during the pandemic. Lyle‐Edrosolo et al. (2023) interviewed 5 ‘system chief nurse executives’ and found initiatives to support well‐being were welcomed. These US‐based studies have highlighted some of the characteristics of effective leadership and how support is valued; however again, the varied roles of participants do not focus on the Executive Nurse Director experience specifically.

Riddell et al. (2022) explored how the pandemic demanded extensive work with Australian executive directors of nursing and midwifery, finding their work involved managing rapid and relentless change that required a multifaceted approach, as well as revealing unintended consequences such as negative outcomes and mixed emotions for nurse leaders. They also highlight how the pandemic resulted in the removal of usual constraints from historical practices and constraints, with new ways of working, opportunities for innovation and stronger working relationships emerging. This study indicated similarities in the role of the Executive Nurse Director in the UK, although the healthcare context differs. Commonalities in experience internationally are found in studies by Romeu‐Labayen et al. (2022), highlighting how nurse leaders in Spain experienced tensions between protecting staff and managing scarce resources, including sparse personal protective equipment (PPI). Discrepancy was also found in staffing levels, with high patient demand combined with high levels of staff sickness. Freitas et al.'s (2021, 2022) interviews with ‘tactical nursing leaders' in a Brazilian hospital, found that challenges were exacerbated by the unexpected and unknown, noting clear and constant communication and proximity to other staff as important, also highlighted in studies of nurse managers by Takemura et al. (2022) in Turkey, Hossny et al. (2022) in Egypt and Daradkeh et al. (2022) in Quatar.

From the international evidence on the varied nurse leader role, suggestions and strategies for the future in supporting nurse leaders and managers during major crises, include targeted support for inexperienced leaders (Holge‐Hazelton et al., 2021), encouraging close and supportive relationships between colleagues (Monroe et al., 2022) and developing disaster policies and increasing emergency resources (Freysteinson et al., 2021; He et al., 2022). Broader recognition of nurses' contributions (Joslin & Joslin, 2020), providing emotional health support and supporting nurses' recovery (Langan et al., 2022) as well as prioritizing policies that support resilience and reduce burnout (Montgomery & Patrician, 2022) are also suggested.

Considering the international literature, it is evident that whilst there has been an increased interest in the experiences of higher level nursing leadership, the evidence remains mixed in its focus and often includes varied levels of management. Whilst these may have similarities and are helpful, the specific nature and responsibilities of the Executive Nurse Director role suggest distinctive requirements, and experiences may provide further insights. The unique context of high‐level nurse decision makers during a public health crisis indicates a need to explore this role further, as an under‐researched group and the potential to inform future professional preparedness. With the specific United Kingdom (UK) Executive Nurse Director role experience unexplored, we set out to gain insight into the experiences of Executive Nurse Directors during the pandemic and to identify leadership strategies utilized. Adding to the international evidence base, we hoped to add a valued contribution to the development and support for future executive‐level nurses and raise awareness, support the development and preparation and inform the debate around the role of the Executive Nurse Director. Effective leadership is critical during all crisis situations. However, there is limited empirical evidence about the role, preparation and experiences of nurse leadership at the executive level during public health crises in the UK and the longer‐term implications.

## 3 THE STUDY

### 3.1 Aims

The project explored the challenges experienced by Executive Nurse Directors in England and Wales during the COVID‐19 pandemic. Understanding the role of executive nurses during a public health emergency was intended to inform future nursing leadership strategies. The research questions were:

- What were the experiences of nurse leaders during and through the pandemic?
- What were the barriers and enablers for these nurse leaders' decision‐making strategies during the pandemic crisis?
- In the wake of the pandemic, what are the priorities, strategies and actions for recovery and learning for the future of leadership in nursing in other public health crises?

### 3.2 Methods

#### 3.2.1 Design

This was a qualitative study, using semi‐structured interviews. A reflexive thematic analysis was applied using Braun and Clarke's (2021) framework whereby initial codes were identified and themes developed and refined. The position of the research team aligned with a constructivist epistemology, situating the approach of the research and the analysis of data in the context of the values and experiences of the researchers (Mann & MacLeod, 2015).

#### 3.2.2 Theoretical context

A descriptive qualitative design was employed which allowed an in‐depth exploration and interpretation of experience with a focus on leadership (Denzin & Lincoln, 2018). We draw on aspects of crisis and situational leadership theory which consider the complexity of the issues and unchartered experiences of the leaders (Riggio & Newstead, 2023).

#### 3.2.3 Participants

Purposive sampling was used and to recruit this specific group, the Chief Nursing Officers in England and Wales were contacted with requests to share the study's aims with national fora and networks for Executive Nurse Directors. Social media networks were also used to advertise and engage with wider interested groups and the research team members also used their professional networks to circulate information about the study. All respondents who matched the inclusion criteria were included in the study. Twenty‐one participants were recruited in total, 14 worked in England and 7 in Wales. All participants were executive‐level nurse leaders in the NHS during 2020 when the pandemic was most prominent in the UK (see Tables 1 and 2). Some of the participants have since moved on to new roles both within and outside of the NHS since 2020.

**TABLE 1:** Inclusion and exclusion criteria.

Inclusion	Exclusion
Working at an Executive Nurse Director level during the COVID‐9 pandemic	Not working at an executive‐level role during the COVID‐19 pandemic
Working in an NHS Trust or Health Board during the COVID‐19 pandemic in the UK	Not working in an NHS Trust or Health Board during the COVID‐19 pandemic in the UK

**TABLE 2:** Participant characteristics.

	Executive Nurse Directors *n* = 21
**Executive Nurse Director**	
Male	8
Female	3
**Geographical location during the pandemic**	
Wales	7
England	14
**Time in executive leadership role**	
1–5 years	10
5–9 years	8
10+ years	3

#### 3.2.4 Data collection

Interviews were conducted between April 2023 and August 2023 by three members of the project team, all are Doctoral level researchers, two experienced nurse academics male and female, and one experienced female Social Sciences researcher. Flexible times were offered to suit the participant's busy schedules. Interviews were conducted around open‐ended interview questions and used topics to prompt the participants, examples are included in Table 3.

**TABLE 3:** Examples of prompts and questions.

Examples of open‐executive nurse director questions
What was your role during the pandemic in terms of the executive board and wider? What were some of the key leadership decisions you were involved in during COVID? What were your professional and personal priorities during the pandemic time and were there tensions between these? How were decisions communicated in the organization from Board level outwards? What was your approach or philosophy of leadership before the pandemic? Communication with staff – do you think it was effective and successful in keeping staff fully informed and supported? How do you feel that nurses contributed to decision‐making in your organization and wider? In the wake of COVID‐19 how is the service recovering from the pandemic and what are the priorities for the future? Is there anything more you would like to add?

All interviews were conducted online via Teams, using Teams record and transcription facility. Auto‐generated transcriptions were checked by two interviewers with the audio recording for accuracy, corrected where required, and were then anonymized. The interviews lasted from 50 to 80 minutes. The confidentiality and anonymity of participants was ensured through use of numbering and secure data management. Informed consent was gained, and participants were reminded that they could withdraw from the study. In recognition of the sensitive nature of the project, strategies for potential emotional distress were employed including offers to pause or stop the interview and signposting to support sources within their organizations and other support services. The potential bias and positionality of the researchers were discussed at team meetings throughout the research process to uphold study integrity.

#### 3.2.5 Data analysis

**FIGURE 1:**
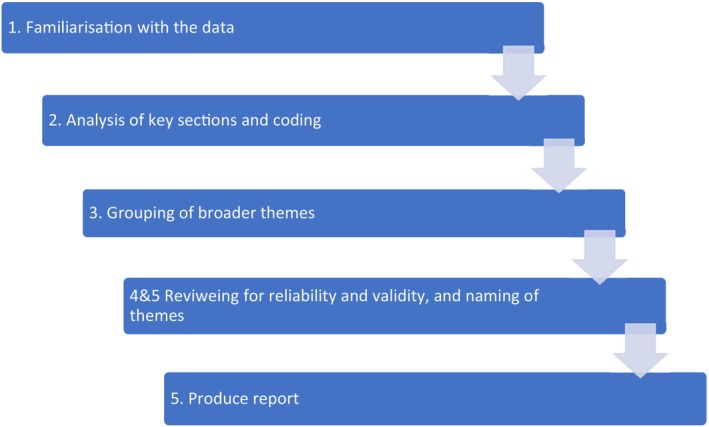
Overview of thematic analysis (Braun & Clarke, 2021).

Thematic qualitative analysis was used to identify and report prominent patterns within the interview data. An inductive process of analysis was based on Braun and Clarke's (2021) six steps of reflexive and thematic analysis (Figure 1.). Each transcript was read several times by three team members which enabled familiarity with the data (stage 1) and an iterative process of analysis, highlighting key sections and adding comments. Microsoft Word was used, rather than a qualitative data analysis package, to ensure accessibility for all team members. The team met to generate initial codes from the data (stage 2). The transcripts were then notated according to these codes, and similar examples were brought together in a separate analysis document. The codes and associated data were then grouped according to broader ‘themes’ (stage 3). The team reviewed these themes to ensure they represented the complete data set (stages 4 and 5). The final stage (stage 6) produced a report, where analysis and synthesis continued, examples of text were selected, the literature consulted again and the research questions addressed.

#### 3.2.6 Ethical considerations

Ethical approval was granted by the University Research Ethics Committee in December 2022. Data were stored according to the University guidance and the UK Data Protection Act (2018). Interview transcripts were anonymized, and identifiable specific organizations and names removed.

#### 3.2.7 Rigour and reflexivity

The data were coded and categorized by three members of the research team to ensure consistency when identifying themes within data. Rigour was achieved by each team member independently reading the transcripts several times, and then meeting frequently to reflect, discuss and triangulate analysis (Morse, 2018). To ensure the confirmability, transparency and auditability of findings, analytical codes and themes were clearly linked to verbatim extracts from transcripts, supporting the reliability of our study. Member checking was considered during the continuous process of interviews and analysis (Lincoln & Guba, 1985), with clarification sought if needed.

In taking a reflexive approach throughout the research process, the research team committed to exploring personal values, professional experiences and knowledge, forcing personal cross‐examination at each juncture. In questioning our own paradoxes and contradictions, as well as how we shaped our interaction with the participants, we hoped to achieve critical subjectivity (Denzin & Lincoln, 2018).

### 3.3 Findings

Six themes were identified from the data which together address the study's aims and objectives:

- Tensions, and adaptive response to personal leadership styles.
- Uncertainty and support at board level.
- Responding to national political decision‐making.
- Personal and emotional impact of the role and the sources of effective support.
- The voice and public profile of nursing.
- Lessons learnt and strategies for future leadership development.

#### 3.3.1 Theme 1. Tensions and adaptive response to personal leadership styles

Participants described the tensions of functioning in a crisis, or a ‘permanent major incident state’ (11). In this context, decisions were being made constantly and rapidly, with either little or rapidly changing information on which to base decision‐making, necessitating the need for a more adaptable and situational style of leadership. This was particularly challenging for leaders who referred to themselves as ‘values‐based’, ‘compassionate’ and ‘collaborative’ in their approach to leadership. The need to adopt a hierarchical ‘command and control’ and a more transactional leadership approach became a necessity in response to the urgency and rapidity of messaging that changed continually from the government. Awareness of this tension was evident as some participants recalled their collaborative leadership style prior to, and the contrasting approach adopted during the pandemic. Participants noted that staff appreciated direct decisions and transactional leadership due to the uncertainty and fear of the conditions in which they were working. However, participants also believed that demonstrating compassionate and values‐based leadership was still possible and imperative to maintain morale and demonstrate empathy with staff:

So, my philosophy was, although I said that about command and control, I'm very mindful of compassion and that people were doing their very best in very difficult circumstances, so try to be visible, I did try to be visible. (09)

Most participants expressed that ‘command and control’ approaches clarified communications and supported staff in understanding and completing the necessary changes in clinical practice. Some felt that the operational freedoms facilitated by the crisis supported an inherent and authentic style of leadership, which was otherwise obscured by bureaucracy and tasks that were now suspended.

When the pandemic hit, it was all hands to the deck. No don't ask for permission….it was low, low bureaucracy, high trust. So, I flourished in that environment because that played to my leadership style. (13)

Others spoke about restrictions in access to clinical areas, creating a tension for Executive Nurse Directors who believed that leading in a crisis required visibility, collaboration and the breaking down of boundaries. Yet, a transactional approach was also described as welcomed by colleagues in terms of how decisions were made and clearly communicated. Executive Nurse Directors acknowledged that whilst these tensions were inherently unavoidable and difficult to manage, they were also understandable and acceptable in the context of a crisis.

I think from a COVID perspective, it was, you know, there was little room for discussion. A decision had to be made and it was made and then communicated down. And I think, and we've had lots of reflection time about this, I think staff really welcomed that type of style because they were all really concerned about what was unfolding and they wanted somebody to make a decision. (17)

Waiting for last‐minute national guidance from government became the norm during the pandemic, yet there were examples of Executive Nurse Directors acting ahead of guidance, making local decisions based on their own judgement. These included the introduction of mask‐wearing and preventing visiting, as their knowledge and experience guided their decision‐making. Also evident was the support of such decisions by the board, approaching it as a collective, and supporting the Executive Nurse Directors. Although participants acknowledged responsibility for decisions which sometimes invoked negative responses from others, the approach of collective decision‐making by their board was hoped to offer some personal and professional protection for them.

We were beginning to make decisions ahead of any national guidance because we could see what was coming. And I think therein lies a really valuable thing around psychological safety. So, our board and our team, were given the freedom to do what was right, just do the right thing and there were a number of things and number of interventions that we did that actually we'd taken three or four days before national guidance came out because we could. (12)

So, there was always, you know, a very, very close working relationship with other executives as part of the team and we would support each other in terms of decision making. (15)

Despite the challenges of operating in extreme conditions and considerable crisis, there were reported challenges and additional ongoing tensions when returning to normal life and more usual leadership approaches after the pandemic.

It took the operational teams quite a while to get used to not having that very clear direction, where we were very specific about what needed to happen by when, go away and make it happen and they would then work more easily as well across the organization. (01)

#### 3.3.2 Theme 2. Uncertainty and support at board level

Support from the executive board was considered important by the participants and examples of adaptive approaches of leadership and functioning were provided in response to uncertainty and urgency. Boards responded rapidly to the pandemic situation, with most meeting weekly, if not daily, which was different from their normal monthly functioning, and these changes were valued. The primary benefits included offering scrutiny, transparency and ensuring that senior leaders were publicly accountable. Most participants described positive collegial relationships with other executive board members, acknowledged as more established and valued than in usual circumstances, reflecting the shared sense of goals and decision‐making that became crucial during the crisis.

Yes, I think it was very bonding with regard to the executive team, myself and the medical director particularly. I think the pressure was enormous. It was absolutely huge. (08)

However, not all participants felt that the board was united in its approach and style of leadership, highlighting tensions around visibility and understanding.

I suppose it was tough because there were only two of us walking the floor. There was only two of us, who knew really what was going on, who were speaking to staff directly. And I think that was difficult because nobody else on the board had that level of granular insight as to what was happening, as everybody else was just, you know, working from home. (05)

The focus on clinical nursing expertise and the respect for the Executive Nurse Director's professional insight was highlighted whilst other board members were perceived as sometimes lacking specialist clinical knowledge that was key for decision‐making. There was a flattening of the hierarchy and adaptive leadership noted within the board context.

And whilst they [executive team] provided some questioning, at times they were aware that the Infection Prevention Control cell was a specialist element there and so therefore made the decisions. So, they would approve and rubber stamp and give agreement then for it to go out. (13)

#### 3.3.3 Theme 3. Responding to national political decision‐making

Participants reported that a sense of uncertainty prevailed for organisations and staff amidst a lack of adequate levels of information and guidance from the UK Government. They also reported an urgency to make decisions in difficult circumstances as the consequences and outcomes of the virus became realized for staff, patients and families. Whilst organisation and planning were challenging, there was a rapid response by the board leaders to address the emerging crisis through a coordinated approach.

Every bit of guidance we had been waiting for all week would land at about 14:00 o'clock on a Friday afternoon and we'd have to work on it as a network into the Saturday…. The biggest barrier was waiting for guidance nationally. It was so slow at times, and you know, we had to make decisions about all sorts of things that were being delayed because of national direction…. You know, it was left to local interpretation, and I think that caused some barriers, particularly where we wanted to procure things locally. You know, particularly PPE equipment. (11)

The rapid spread and emerging evidence meant that Executive Nurse Directors were expected to make decisions based on rapidly changing guidance. Implementing guidance from Government agencies, which were sometimes confusing and conflicting, could lead to tension.

You could be having a conversation with a member of staff in the corridor about the level of PPE that they had one day. Go back up to the office and find that the advice had changed. And you know, I knew that just half an hour ago I'd given you know, advice to a member of staff that was now wrong half an hour later. (04)

#### 3.3.4 Theme 4. Personal and emotional impact and sources of effective support

The challenges of the executive‐level role had an impact on both the personal and professional lives of the participants, resulting in a significant impact on work/life balance due to long working hours, and less family contact. Participants also unsurprisingly experienced episodes of low mood and exhaustion, with the responsibility of decision‐making and trying to maintain the morale of others.

I think pushing people to the limit and seeing such shocking things I don't think were, I mean, I remember walking into the intensive care unit and just seeing patient upon patient proned, ventilated and…. most of our patients were young. Um, and you, don't forget that. (08)

I left my family. I packed my bags…. and I had no idea when I'd come home. So no, we worked 18 hours a day, most days […] The bit that I found really difficult was the social isolation during that period and not just being away from your family and your support mechanism and arriving in a team that you didn't know anybody at all. (03)

Participants mentioned critical moments which caused them to pause and reflect on the implications with emotional force.

And then I just looked at the team and they were, you know, it was one of those cases. Everybody had seen so much death so much, but this particular young lad. The entire team were just nearly crumbling, and I, as senior person, had to kind of hold it together and then they said, which one of us is going to phone his mother? (08)

And then we have the death of the staff member and it hit us hard, it hit the ward hard. It hit the hospital hard. It kind of ricocheted across (04)

Participants recognized the value in drawing on their professional networks for support, viewing these as safe spaces to seek reassurance and advice from others with similar experiences.

[What nurse leaders were facing] was beyond what you would expect normally. So, there was real pressure. So, there was a real need for that supportive and open culture, which I think we had anyway, but that real permission to share whatever and to be that clear, if you think something's gone wrong, to be able to be open with that, without fear of reprisal. (08)

The sense of vulnerability, togetherness and acknowledgement of the need for self‐care within this emotionally unpredictable context was considered essential. It was supportive because of the sense of being part of a wider network of senior leaders, all with shared values and experience.

#### 3.3.5 Theme 5. The voice and public profile of nursing

Participants discussed perceptions of the profession during the pandemic, responses from the public and contrasting views. Outward appreciation was visible in the media in the early days and gifts of sustenance from the public were appreciated. However, participants felt that at times the difficult position of nurse leaders was not always recognized, for example when implementing national guidance meant preventing relatives from visiting hospitals and care homes. Participants acknowledged that the decisions made sometimes left them feeling both personally and professionally vulnerable and isolated. One participant [06] received threats from relatives after they were prevented from entering the hospital and was told that she was hated. In this example, this was reconciled with the fact that the right decisions had been made which had kept patients safe. Professional vulnerability was also expressed, and careful and detailed documentation was important to prepare for future scrutiny and accountability for the decisions taken. Some participants expressed the belief that the public perceived nurses could cope with the associated emotional impacts and, some felt these had not been addressed.

And the risk that you carry inherently of being a registrant and the privilege of being on that register could be taken away from you if you can't justify and everything. So, we're really good at documenting decision making. (01)

So, the resilience sometimes in trying to look after a lot of people and particularly being a nurse where people have other expectations of you in terms of that perception where you're a nurse, you're caring, you're used to all this sort of stuff, aren't you? You can deal with all that emotional baggage. You can deal with all the fallout, you can deal with the sick and the wounded and everything else, so it was quite interesting about perceptions of people. (21)

The challenges facing the nursing profession were acknowledged and participants believed this had worsened rather than improved since the pandemic. Low morale and a subsequent worsening crisis in staff recruitment and retention were challenges that were compounded further by nurses taking industrial action in the UK. Many were concerned that public perceptions of nursing as an attractive profession were now at risk.

…. we absolutely have to recognise and address, consult how we are going to make the profession attractive again. I think for me that absolutely has to be a priority. We are a leaky bucket as a profession […] it's going back to my earlier conversation around the opportunity and the ambitions for nursing as a profession that is the most respected and the most trusted. (15)

Many felt that whilst nursing had been publicly and politically represented by nursing leaders in the UK, participants were uncertain whether senior nurses had been given the opportunity to influence higher decision‐making at the national level. Some expressed a lack of visible nursing leadership during this time, and a failure to clearly define the nature and value of highly skilled nursing work and its contribution to healthcare. Several participants underlined the importance and risks of senior nurses speaking out, albeit acknowledging that this can be difficult to achieve.

…another good bit of learning from the pandemic is about voice, whether we like to hear that or not, that is what happens and at a senior level the influence of others becomes stronger and stronger. […] We are the leaders of the profession and if we don't feel safe to speak or don't have a route speaking up, then yeah, what? (12)

#### 3.3.6 Theme 6. Lessons learnt and strategies for future leadership development

The interviews with participants provided an opportunity for them to reflect on their learning from the pandemic experience. Participants discussed the nature of effective leadership during a global crisis, the importance of adaptability, trust in others and in organizational processes. In addition, the need for transparency, honesty in decision‐making and the importance of strengthening and valuing of relationships were emphasized.

Although it was difficult at that time it's created a different relationship for me with that team…. a kind of a bond. We walked that road together and that is completely there, and it's allowed us to then tackle other things like the (nursing) strike actions differently. So, I think you know the relationships are really key. I think that's something, that's definitely very, very apparent. (20)

Acknowledging the complexity of the role of senior nurse leaders, and all the associated uncertainties and nuances during this time, was evident. Future support and development also featured an acknowledgement that a more structured approach to developing nurse leaders at the strategic level was needed, with opportunities to learn and experience that level of nurse leadership, which was sometimes lacking. Advice was offered for future leaders to develop those skills.

Thinking about how you can help leaders, nursing leaders and midwifery leaders of the future. Go look, see. And by that, I mean get out into the wider world, have the opportunity to go visit other services, meet with other leaders to see what their style is, observe board meetings in other organisations.’ (01)

I think we shelter a lot of early nurses in their career from a lot of that. And then we wonder why they're not able to step up and do that when they join us, because they've had no exposure and no learning unless they've had good life experience. (21)

Necessary changes resulting from the pandemic had provided some with a sense of freedom to move away from targets and bureaucratic constraints, and whilst some participants acknowledged ways of working had since returned to pre‐pandemic structures, one recommended for the future for nurse leaders was to preserve the freedom, creativity and the breaking down of organisational barriers that were facilitated through this crisis.

## 4 DISCUSSION

This research offers an important lens on the experiences, approach and learning from those nurses who were in executive‐level leadership and decision‐making positions during the COVID‐19 pandemic in the UK. Responding to calls for more evidence to be made available on the experiences of directors of nursing to strengthen the role, this study aimed to explore the complex and challenging role of being an executive‐level nurse during the pandemic (Fowler & Robbins, 2022; Horseman et al., 2020).

We analysed the experiences of Executive Nurse Directors during the pandemic and six themes were developed from the findings as set out in Table 4. Our findings highlighted the leadership challenges that occurred at the personal, executive board and national levels as well as the uncertainties, tensions and adaptive leadership approaches experienced by these participants. Many viewed leadership skills as being more essential than ever during the recent crisis and highlighted the challenges of constantly changing national guidance and communications and translating these into practice in the local context. The executive board's role in supporting the decision‐making capacity of executive‐level nurses, the frequency of meetings and a collective pressure to respond to the impact on staff and patients were reported as having impacted positively on the role at this time. The flattening of the hierarchy, when it occurred, and the sense of shared influence within the executive board was in contrast, with Kelly et al.'s (2016) pre‐pandemic study of these roles.

**TABLE 4:** Research themes.

Tensions, and adaptive response to personal leadership styles Uncertainty and support at the board level Responding to national political decision‐making Personal and emotional impact of the role and the sources of effective support The voice and public profile of nursing Lessons learnt and strategies for future leadership development

Research on crisis leadership (Riggio & Newstead, 2023) often includes the re‐organization of roles, due to high necessity, where skills are re‐prioritized in terms of the knowledge and experience required. This adaptive approach was particularly evident where infection control experience and knowledge of the staff and teams took priority.

Whilst previous studies have identified the challenges of decision‐making, in the context of the pandemic, our participants described how they were impelled to make complex decisions ahead of guidance, from the wearing of masks, to preventing visitors and closing wards. These were considered critical lifesaving decisions, yet sometimes resulted in negative responses from the public, highlighting the personal vulnerability of nurse leaders in this situation.

However, our participants felt empowered through a shared sense of support and ownership of problems, identified as an enabler for effective leadership and was reinforced by how the executive board had functioned. Throughout, Executive Nurse Directors remained aware of their ultimate professional vulnerability and responsibility in decision‐making and had concerns about post‐pandemic consequences both for themselves and for the profession as a whole. We have identified a leadership paradox here, where the unique concentration of national focus on infection control and restrictions simultaneously enabled a sense of freedom and innovation, with local opportunities unrestricted by usual decision‐making conventions, hierarchies and bureaucracies.

Participants expressed how the pandemic had introduced changes that enabled nurse leaders to maintain a singular focus on care and safety for patients, without additional concerns such as the meeting of targets, financial restraints, or excessive administrative tasks, and where the giving and taking of advice and orders became accepted and normalized. These changed circumstances had supported a stronger nurse leadership voice, enhanced by greater opportunities for collaboration and cooperation across departments and hierarchies, and a stronger sense of shared respect towards all staff. Participants were quick to compare those times with the current landscape of nursing leadership. ‘Clapping for carers’ across the UK and the donation of gifts offered momentary solace and were a welcome display of public acknowledgement of the work and risk to nurses. However, there is now a recognition that much more was, and is, needed, and a shift away from angelic narratives to strengthen the presence of nurse leadership, as supported by Bennett et al. (2020) and James et al. (2023) is needed. This study correlates with Riddell et al. (2022) who also identified the strength of nurse leaders and the effect of innovation by the removal of barriers which enabled a clearer focus on patient care and stronger working relationships. Notably, we found participants felt that their voice was being respected and listened to by other members of the board, in contrast to previous studies where they had sometimes struggled to have the same influence (Kelly et al., 2016).

There are also similarities with the findings of Kelly et al. (2023) such as status and the responsibility of the executive nurse role, the influence that executive nurses have beyond their role, concerns about being held personally liable for organizational failure, and the value of coaching and mentoring. These similarities suggest a strong sense of continuity in the call to better understand this role, particularly by developing a greater understanding of its demands and complexity (Horseman et al., 2020). The role of the nurse leader has been identified as especially critical during a health crisis (Losty & Bailey, 2021). We found that nurse executives were highly aware of this responsibility, not just towards keeping patients and staff safe, but in terms of presenting effective examples of leadership, of being open and honest and creating the right conditions for nursing staff to feel supported and sustained.

The experiences of emotional effort and implications of such ‘pandemic emotion work’ (Dowrik et al., 2021) are also important to consider here, particularly considering increasing recognition of the moral distress experienced within nursing and its relationship with current constructions of resilience (Fitzpatrick et al., 2022). Nurses have experienced an emotional burden as a result of the pandemic and there was an acknowledgement that this had yet to be appropriately addressed. If executive‐level nurses' contributions and learning is to be valued further, then opportunities for expression and support should be provided, along with structured succession planning based on their learning. Lack of opportunities to explore emotional response and reasoning from unique leadership experiences, such as during a global health crisis, will only negate and disregard important pragmatic learning (James, 2023).

In conclusion, the project explored the experiences of Executive Nurse Directors in the UK during and through the pandemic. We found that nurse leaders experienced challenges at all levels, and they felt uncertain about the future and the immediate strategy. Nurse leaders experienced an emotional burden and heightened awareness of their role and the consequences to professional status. The pandemic brought challenges to values‐based leadership styles and demanded adaptable approaches. Nurse leaders expressed extensive concern for staff and patients, whilst experiencing personal sacrifice and distance from family and support. They valued their peers and other nurses in executive roles network amplifying the particular pressures of this high‐level role.

We have identified significant barriers and enablers for nurse leaders' decision‐making strategies during the pandemic crisis. Enablers Included support from peers and the executive board in supporting decision‐making; regular meetings and a collective pressure to deliver quality care in a crisis; the flattening of the hierarchy; and the removal of usual processes allowed innovation. Barriers Included changing public perception and portrayal in the media; uncertainty from decision makers nationally and changing communication; emotional burden and lack of support.

Finally, we identified the priorities, strategies and actions for recovery and learning for the future of Executive Nurse Directors and wider senior leadership in nursing in other public health crises. These included recognition of the impact of nurse leaders and their effect on innovation, enabling a clearer focus on patient care and fostering stronger working relationships. In addition, we identified the need for a better understanding of the executive role as well as succession planning; greater visibility and the accessibility of executive role models who can present examples of effective nurse leadership as well as greater awareness of the dynamic challenges that these roles will face in the future.

### 4.1 Strengths and limitations

This study has provided a unique perspective of the leadership experience of executive‐level nurses during the pandemic in the UK. It complements a small but important body of work focusing on the role of executive nurse leaders working at this level. Participants were keen to contribute and to have their experiences heard.

Although the numbers recruited compare well with similar qualitative studies, only a small number of nurse executives took part. This highlights recognized difficulties for research recruitment in high‐level executives. O'Reilly and Parker (2012) provide guidance for concerns that full saturation of data capture can never be achieved due to the organic nature of the human experience. Transparency of the epistemological and methodological stance guided the decision‐making process within the research and the challenges of time and opportunity for interviewing this level of professional is acknowledged as a limitation. A further limitation is the focus in this study on Wales and England, and we encourage future international comparative research. We also stress a sense of urgency, to conduct such research as some nurses who led during the pandemic remain in nursing.

### 4.2 Recommendations

By understanding the experience of nurse leaders during the pandemic, this article builds on previous research which has identified how nurse leadership can be better supported in the future and some findings apply to the nature of the Executive Nurse Director role both in the UK and globally. Participants provided many suggestions about how nurse leadership could be better supported prospectively to ensure their lived experience had an impact on future pandemic planning. These include encouraging broader learning from recent public health crises for the preparation of aspiring leaders, investment in structured development programmes and access to mentorship. Opportunities to shadow Executive Nurse Directors including observation and attendance at board meetings to gain an understanding of how decision‐making takes place at the board level are recommended. Opportunities for nurses to reflect on their experiences during the pandemic have provided key learning about the potential to be creative in terms of this role and its functions, as this had clear benefits for some. Their experience during the pandemic highlighted how elements of normal working patterns at the executive level in NHS organizations can act as barriers, which restrict opportunities for creativity and innovation.

In addition, there is a need to support the voice and visibility of executive‐level nurses at a local and national level, as well as encouraging innovation and creativity within the constrictions of heavy workloads and bureaucratic systems. More than ever, there is a need to question how executive‐level nurses can enhance the profile of nursing as a profession and make visible the achievements and possibilities of this career pathway to encourage a future executive workforce. Many of the participants expressed personal benefits in contributing to this project, by being given an opportunity to talk about their experiences, to reflect on their responsibilities during the pandemic, and the potential to shape future nurse leadership.

## 5 CONCLUSION

Executive‐level nurses contributed significantly to the successful leadership of health services and decision‐making during the COVID‐19 crisis. This study explored the experiences of nurses working at the executive level and identified recommendations for strengthening this role in the future. However, further research into nursing leadership focused on at this level that will question how best to support future aspiring nurse executives is needed, thereby acknowledging the contribution and wealth of expertise that already exists, as well as ensuring a voice and legacy for the profession.

## AUTHOR CONTRIBUTIONS

AHJ, RD, AJ, DW, DK: Made substantial contributions to conception and design, or acquisition of data, or analysis and interpretation of data; AHJ, RD, AJ, DW, DK: Involved in drafting the manuscript or revising it critically for important intellectual content; AHJ, RD, AJ, DW, DK: Given final approval of the version to be published. Each author should have participated sufficiently in the work to take public responsibility for appropriate portions of the content; AHJ, RD, AJ, DW, DK: Agreed to be accountable for all aspects of the work in ensuring that questions related to the accuracy or integrity of any part of the work are appropriately investigated and resolved.

## CONFLICT OF INTEREST STATEMENT

No conflict of interest.

### PEER REVIEW

The peer review history for this article is available at https://www.webofscience.com/api/gateway/wos/peer‐review/10.1111/jan.16329.

## Supporting information


Data S1.

## Data Availability

Data available on request from the authors.
